# Motor vehicle crashes on tribal reservations: mapping and statistics

**DOI:** 10.1186/s40621-021-00361-7

**Published:** 2021-12-20

**Authors:** Jordan M. Vandjelovic, Darcy Merchant

**Affiliations:** Division of Environmental Health and Engineering, Indian Health Service, 2900 4th Ave. N, Suite 407, Billings, MT 59101 USA

**Keywords:** American Indian/Alaska Native, Motor vehicle crashes, Mapping, Injury, Fatality

## Abstract

**Background:**

Motor vehicle crashes (MVC’s) in the American Indian/Alaska Native (AI/AN) communities account for 43% of unintentional injury deaths. This article introduces MVC data and geographic information system (GIS) mapping for tribal reservations.

**Methods:**

Utilizing a sample of Montana Department of Transportation (DOT) data for the Flathead reservations to calculate frequencies and proportions of crash types (i.e., property damage or no-injury, injury, fatality or unknown), while also mapping these data to provide a cross-sectional snapshot of MVC’s.

**Results:**

Overall, 515 MVC’s occurred for years 2016 through 2018, with no-injury, injury, and fatality accounting for 72.2%, 24.9% and 1.8% of all crashes, respectively, with the number of MVC’s ranging up to 30 per square mile.

**Conclusion:**

Examining DOT data and utilizing it for visual representation of MVC’s can be used as an additional source in uncovering patterns and trends on Tribal reservations and supporting MVC prevention efforts.

## Background

Motor vehicle crashes (MVCs) are one of the leading causes of death in the USA and account for 43% of all unintentional injury deaths among American Indian/Alaska Native (AI/AN) communities (Centers for Disease Control and Prevention (CDC) 2020; Indian Health Service 2020). For years 2008–2010, the Indian Health Service (IHS) all Areas age-adjusted MVC death rate average was 36.0 per 100,000 population, with the Billings Area having the highest (65.8) rate (Indian Health Service 2020).

The IHS has focused much of its work and data on general epidemiology and clinical outcomes, with little information being gathered on geospatial displays and risk factors.

State Departments of Transportation (DOTs) have highlighted the importance of having comprehensive crash maps as critical components of safety data management and highway safety plans. Similarly, MVC maps can assist Tribal governments, programs, stakeholders and partners in identifying hot spots of high risk roadways, crash severity and contributing risk factors to inform policy, program planning, and evaluation (Qin et al. 2013; Musa et al. 2013; Clarke et al. 1996).

We applied these techniques to the Billings Area tribal reservations and present the findings here. This study uses tribal MVC data and geographic information system (GIS) methods to demonstrate the potential value of geographic visualization as a highway safety tool for tribal reservations.

## Methods

We used existing data and descriptive techniques to accomplish the study aims.

### Population

We examined a sample of MVC on the Flathead reservation within Montana.

### Crash data

We obtained MVC data through requests submitted to the Montana DOT for years 2016 through 2018, the most recent years available. Records were included in the study only if the crash occurred on the reservation property and within the boundaries of Flathead, Lake, Missoula or Sanders counties. Person-level data only included motor vehicle occupants and excluded pedestrians or other individuals. The population studied was inclusive of AI/AN only, which were selected using the Race variable as originally recorded in source documents.

Injury variables in the original DOT dataset were combined to derive the variable Crash Type with three categories: no-injury, injury and fatality (Table 1) for descriptive statistics and map displays.

**Table 1:** Criteria for derived variable: crash type

Values for derived variable: *Crash Type*	Criteria for inclusion of MTDOT variable: *Injury Status Type*
No-injury	No-injury
Injury	Possible injury; non-incapacitating injury; Incapacitating injury
Fatal injury	Fatal injury

### Data analysis

Frequencies and proportions for MVCs by the variable Crash Type. Some data were missing for the variable Crash Type and were therefore excluded for the purposes of this study. All statistical analyses were performed using SAS 9.4.

To map the data, we used ArcMap (http://www.esri.com/software/arcview/index.html), using the XY (latitude/longitude) coordinate variable, to provide a cross-sectional snapshot of MVCs related to Crash Type within the Flathead Reservation. The mapping process begins with a base map that includes layers showing interstate highway systems, major state highways and local roadways, upon which displays of individual crash points and thematic groupings were overlaid. Additional data presented on the maps include summary variables such as the number of injuries per crash.

Thematic maps and gridded shapefiles (files containing vector data constructed of points, lines and polygons) were created with the cell width and height set to 1-mile by 1-mile area, corresponding to specific regions of each reservation. Crash variable attributes corresponding to the gridded shapefiles were summed and then thematically displayed in square mile polygons to include the number of MVCs that have occurred within each polygon.

## Results

During the years 2016 through 2018, there were 515 MVCs involving AI/AN people on the Flathead reservations, being mapped to identify clusters of data.

**Fig. 1:**
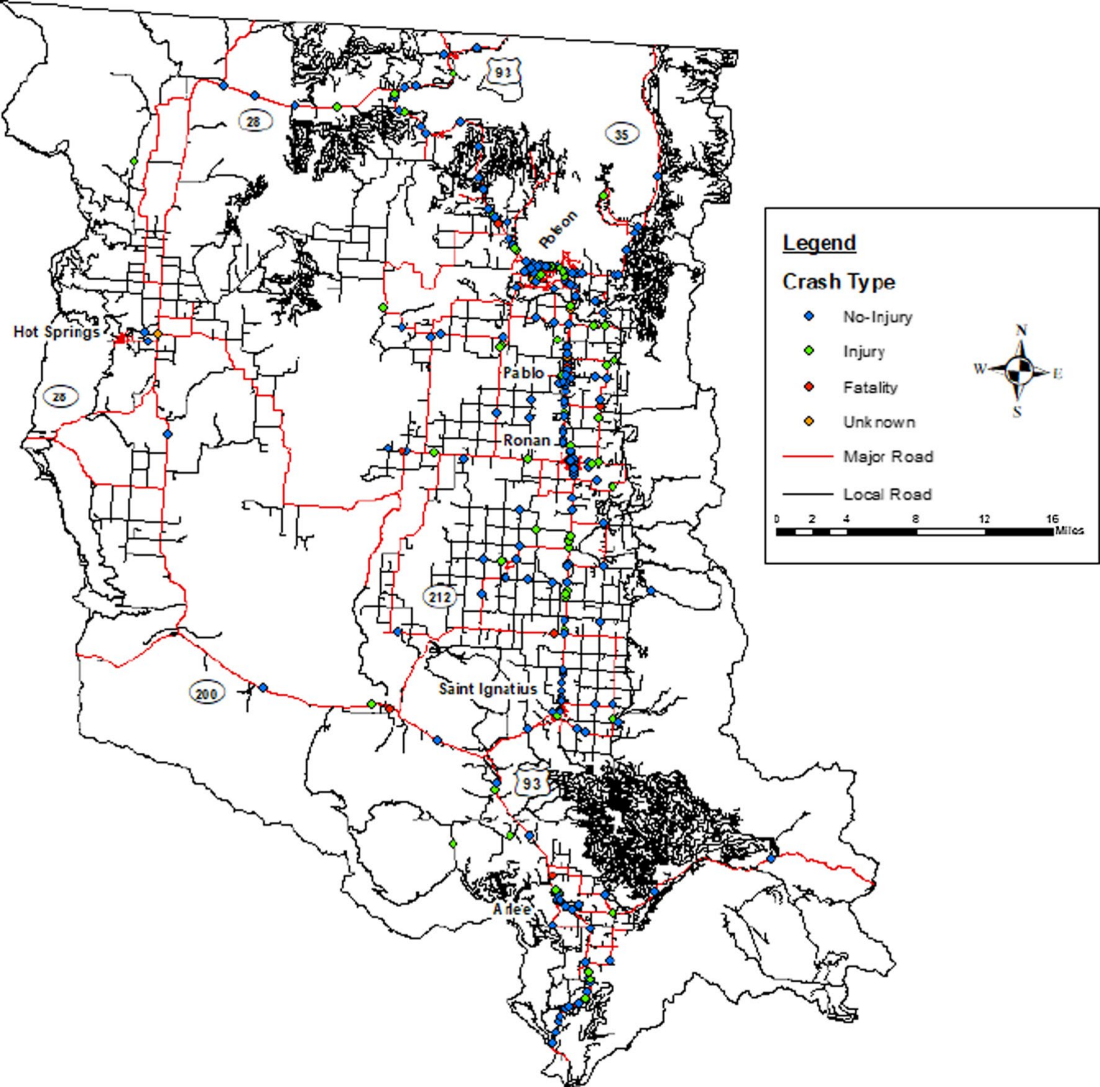
Crash type map of the flathead reservation, years 2016–2018

**Fig. 2:**
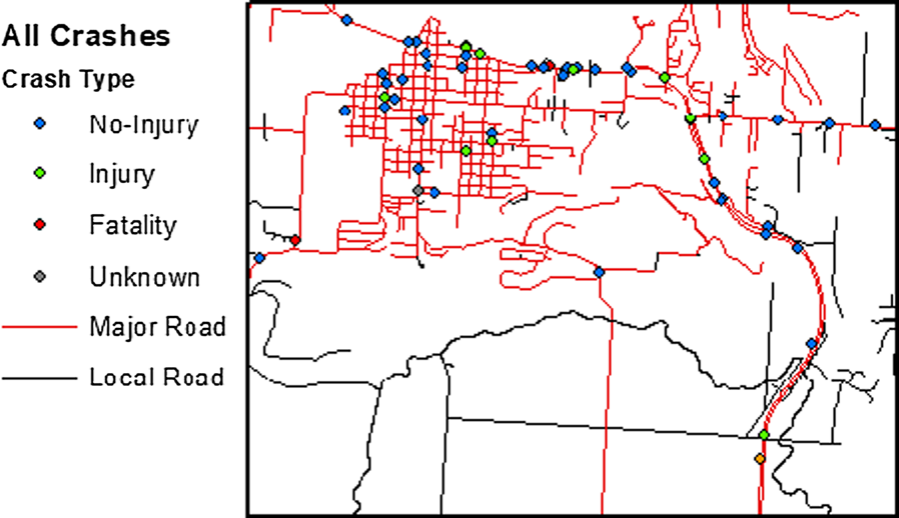
Zoomed in region of roadways on the flathead reservation displaying crash type, years 2016–2018

More than half (72.2%) of all the MVCs resulted in no-injury, with injury and fatal MVCs accounting for 24.9% and 1.8%, respectively (Table 2). Figure 1 shows the distribution of Crash Types across the entire study area, and Fig. 2 displays these same data for a smaller region at a larger scale.

**Table 2:** Proportion of crash type by sex (2016–2018)

Age group and sex		Crash type % (*N* = 515)				
		All crashes	No-injury	Injury	Fatality	Unknown
All ages	Total	515 (100.0)	372 (72.2)	128 (24.9)	9 (1.8)	6 (1.2)

**Fig. 3:**
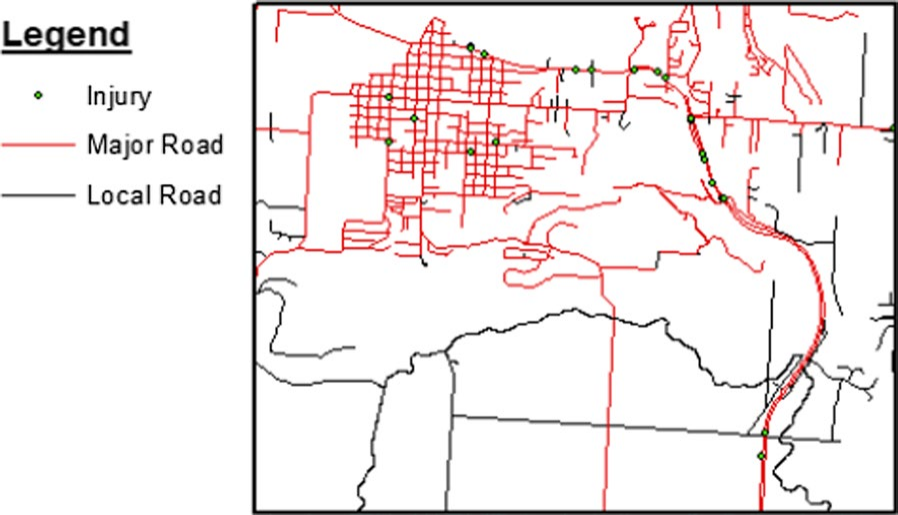
Zoomed in region of roadways on the flathead reservation displaying crash injury events, years 2016–2018

**Fig. 4:**
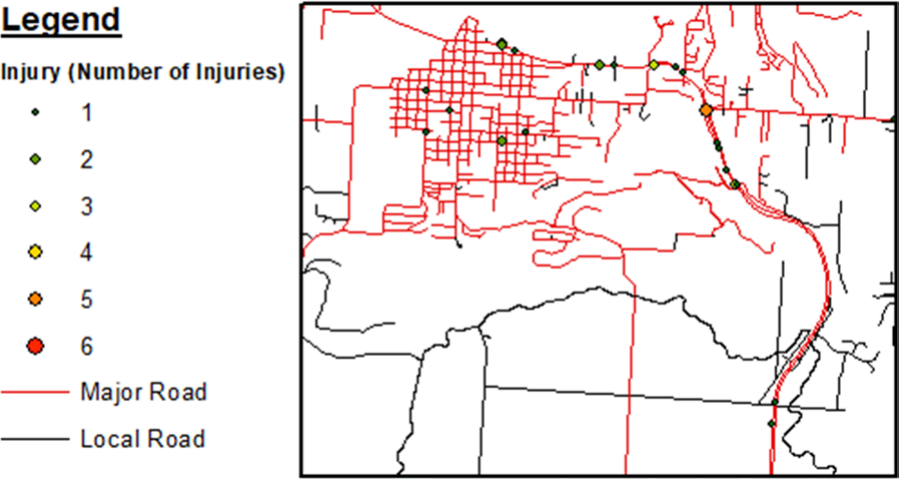
Zoomed in region of roadways on the flathead reservation displaying crash injury events by number of injuries per event, years 2016–2018

Data queries and layering techniques within GIS tools allow the display of subsets of data, making it easier to explore or emphasize items of interest. For example, Fig. 3 displays only MVC events that resulted in injuries, which were isolated and mapped for the same area as in Fig. 2. Substituting the underlying data associated with each plotted point gives additional insight into the area of study. Overall, 61.7% of MVC events resulted in multiple people sustaining injuries, ranging from 1 to 5 injuries per MVC (Table 3; Fig. 4).

**Table 3:** Injuries per motor vehicle crash (2016–2018)

Injury (count per crash)	Frequency (%)
1	49 (38.3)
2	34 (26.6)
3	18 (14.1)
4	18 (14.1)
5	9 (7.0)

**Fig. 5:**
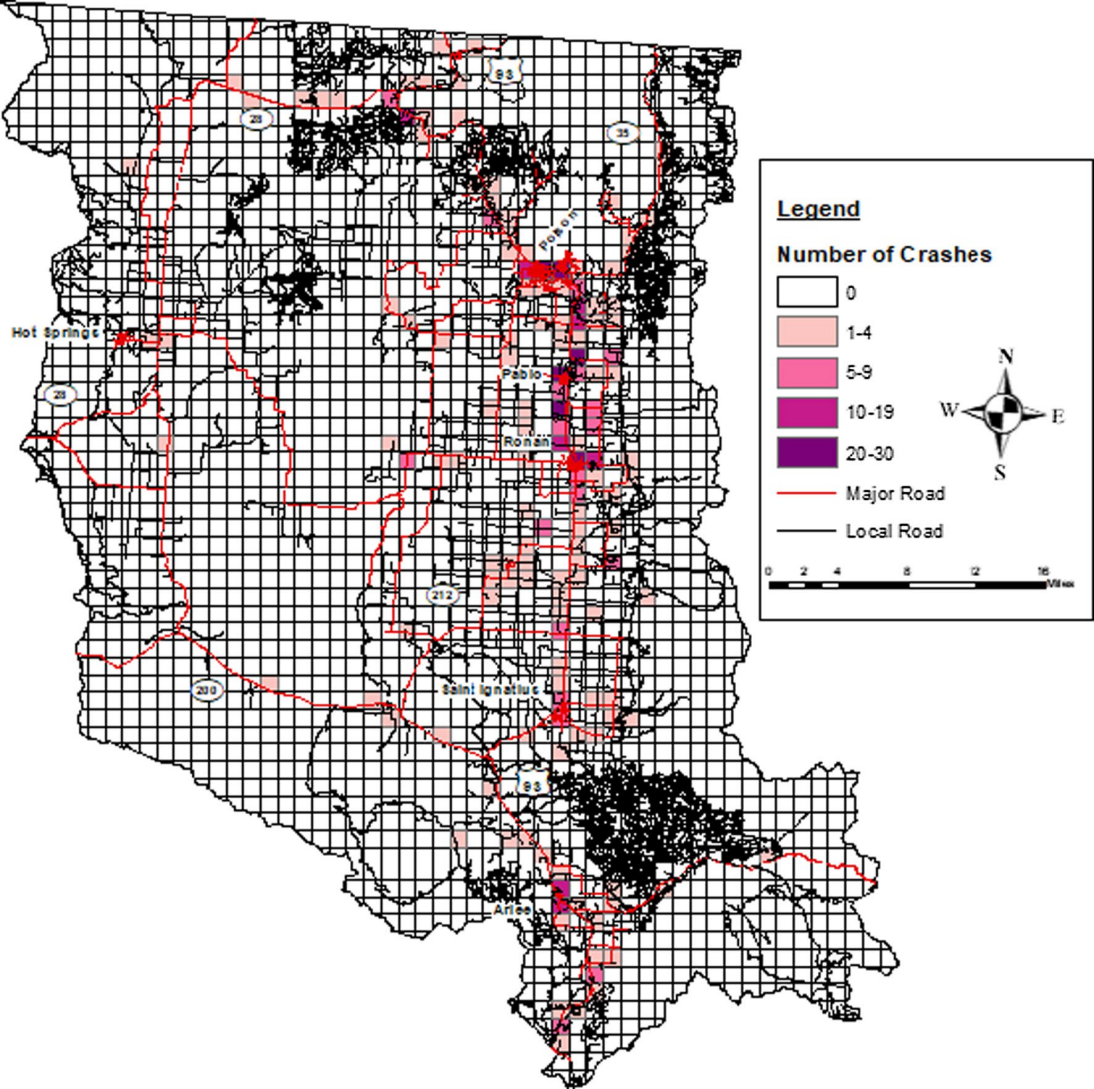
Flathead reservation thematic display of the number of crashes per 1 square mile, years 2016–2018

**Fig. 6:**
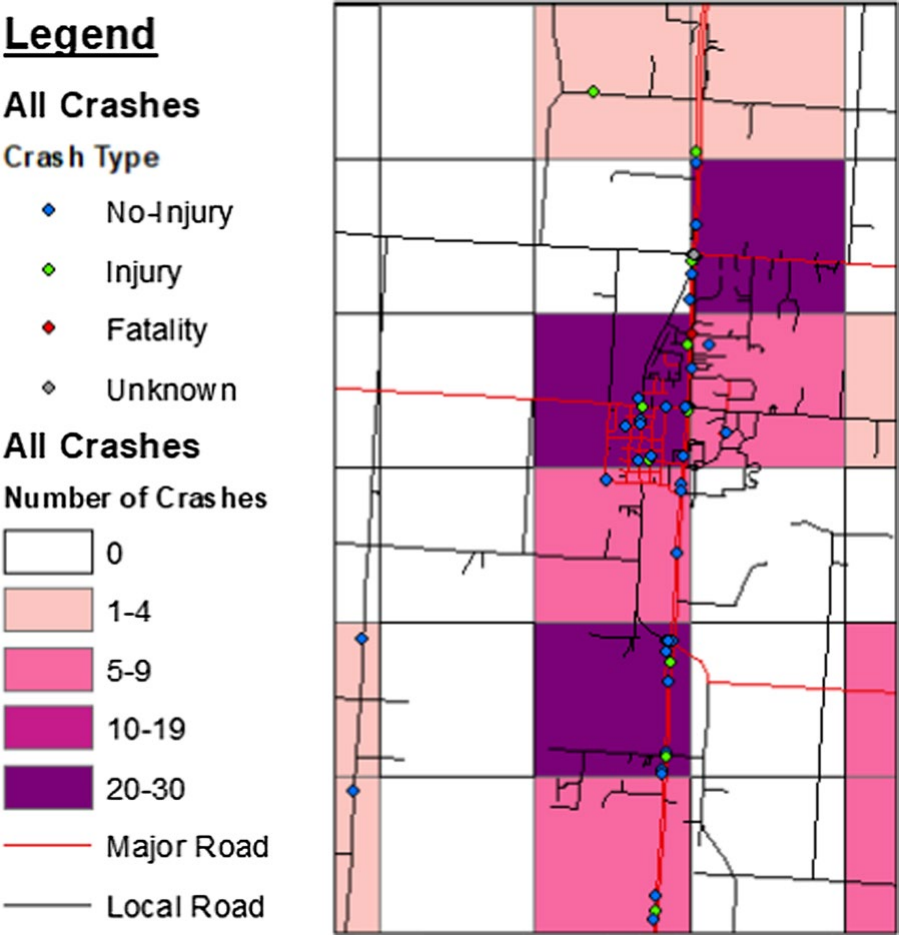
Zoomed in region of roadways on the flathead reservation showing thematic display of the number of crashes per 1 square mile and crash type, years 2016–2018

The total number of MVC injuries and fatalities per 1 square mile segment varied, ranging from 0 to 8 injuries and 0–1 fatalities. Overall, the number of crashes of any Crash Type per 1 square mile ranged from 0 to 30. These can be represented as thematic groups across the entire study area (Fig. 5) and/or focus in on a smaller areas displaying additional crash event data for a more comprehensive view of MVCs in a specific area (Fig. 6).

## Discussion

Using DOT data and basic GIS mapping techniques, further examination of Crash Type and crash cluster locations can provide additional insight into the epidemiology and outcomes of MVCs that may be useful to the IHS.

The findings from this study show the proportion of injury and fatalities, as well as identify areas with high numbers of MVCs. Mapping areas with clusters of MVCs using additional point information such as Crash Type, number of injuries or fatalities involved and summarizing data using thematic displays can combine multiple data elements to identify and characterize areas of high MVC frequencies.

Data and maps such as these can be used to support injury prevention activities, advocate for allocation of funds, aid in program evaluation and implementation as well as pursue changes to policies and laws. Tribal transportation programs continue to partner with local and state agencies to produce road safety audits (https://www.mdt.mt.gov/visionzero/plans/tribal-plans.shtml), to support tribal safety and obtain funding for road improvement projects (Musa et al. 2013; Clarke et al. 1996).

Data and maps similar to these can be utilized to further research evaluating high MVC death rates in the AI/AN population. Examining additional demographic, contributing factors (i.e., age, sex, involvement of drugs or alcohol, time of day, etc.) and other variables provided in DOT data, such as the number of injuries or fatalities per crash and environmental factors at high MVC frequency locations, provide multiple avenues for future scientific inquiry.

This study has several limitations and serves primarily as an introduction to data and mapping for tribes. Due to the vast uses of DOT data and GIS mapping, this study was limited to expanding on additional data, variables and map displays. The quality and completeness of DOT data from reported crashes may include inaccuracies due to missing data (i.e., result of Crash Type due to hit and run), incorrect or inaccurate crash location information, poor base maps, and limited resources and techniques for crashes off state roadways (Qin et al. 2013). Tribal sovereignty must be considered as part of the response to MVCs and in reporting by different agencies (i.e., tribal police and Bureau of Indian Affairs) that may not report to state DOTs. These data did not assess the reliability and inclusion of data for the AI/AN race, with misclassification of race varying greatly through the U.S. (Indian Health Service 1996; Jim et al. 2014).

## Conclusion

The goal of this study was to apply GIS techniques to MVC data as an introduction to the value of geographic visualization as another means to support epidemiological and clinical outcome research used by IHS in support of MVC safety on reservations locally and nationally.

Data sources containing valuable information, such as DOT crash data and others, can be considered when investigating MVCs on Tribal reservations in addition to data currently being used. Furthermore, utilizing GIS for MVC’s and other events can assist in uncovering patterns and trends in injury morbidity and mortality, providing both visual impact and data analysis simultaneously. This can lead to confirmatory and explanatory data solutions to a variety of highway safety questions beyond the traditional research and statistical methods previously utilized by tribes.

## Data Availability

Data made available upon request from the Montana Department of Transportation through contacting mdttdcrequests@mt.gov.

## Abbreviations

MVCs: Motor vehicle crashes
AI/AN: American Indian/Alaska Native
HIS: Indian Health Service
DOTs: Departments of Transportation
GIS: Geographic information system
